# Can Audiometric Configuration Predict Which Patients Derive Greater Speech Intelligibility Benefit from Hearing Aid Fitting?

**DOI:** 10.3390/jcm15197439

**Published:** 2026-09-24

**Authors:** Dae Hyun Kim, Jinsei Jung

**Affiliations:** 1 Department of Otorhinolaryngology, Yonsei University College of Medicine, Seoul 03722, Republic of Korea; ddh0321@yuhs.ac; 2 Won-Sang Lee Institute for Hearing Loss, Seoul 03722, Republic of Korea; 3 Severance Biomedical Science Institute, Yonsei University College of Medicine, Seoul 03722, Republic of Korea; 4 Bio-Centennial Convergence Institute, Yonsei University, Seoul 03722, Republic of Korea

**Keywords:** hearing aid, speech intelligibility, audiologic configuration, maximum word recognition score

## Abstract

**Background**: Although hearing aids improve audibility in patients with sensorineural hearing loss (SNHL), improvements in maximal speech intelligibility vary substantially among individuals, and reliable predictors of hearing aid benefit remain lacking. We investigated whether audiometric configuration predicts speech intelligibility benefit following hearing aid fitting in adults with moderate SNHL. **Methods**: In this retrospective cohort study, 351 adults with newly diagnosed moderate SNHL who were fitted with hearing aids between January 2017 and December 2021 at a tertiary referral center were classified as having down-sloping or flat hearing loss according to the difference between low- and high-frequency hearing thresholds. Speech intelligibility benefit was evaluated using the maximum word recognition score (WRSmax) before hearing aid fitting and at 1 month and 1 year after fitting. **Results**: Compared with patients with flat hearing loss, those with down-sloping hearing loss demonstrated greater immediate improvement in WRSmax at 1 month (10.43 [10.32] vs. 7.18 [9.44] percentage points, *p* = 0.004) and greater cumulative improvement at 1 year (11.00 [10.55] vs. 6.79 [11.79] percentage points, *p* = 0.020). These associations remained significant after adjustment for age and sex (B = 2.506, *p* = 0.024 and B = 3.792, *p* = 0.044, respectively). **Conclusions**: In adults with moderate sensorineural hearing loss, a down-sloping audiometric configuration was associated with greater improvement in speech intelligibility following HA fitting than a flat audiometric configuration.

## 1. Introduction

Age-related hearing loss (ARHL), or presbycusis, is one of the most common chronic conditions affecting older adults worldwide [1]. The World Health Organization estimates that more than 430 million people currently require rehabilitation for disabling hearing loss, a figure projected to exceed 700 million by 2050 [2]. Beyond reducing hearing sensitivity, hearing impairment adversely affects communication, social participation, quality of life, and cognitive health [3]. In addition, hearing impairment has been recognized as a potentially modifiable risk factor for dementia [4,5,6]. In aging populations, hearing difficulty arises from multiple mechanisms, including peripheral auditory dysfunction associated with cochlear degeneration, deficits in central auditory processing along the auditory pathway from the cochlear nucleus to the auditory cortex, and age-related cognitive decline involving executive function, language memory, semantic and episodic memory, and psychomotor processing [5,7,8]. Because no curative treatment currently exists for presbycusis, hearing aids (HAs) remain the primary intervention to improve audibility, facilitate communication, and potentially mitigate cognitive decline in the elderly [4,9].

Sensorineural hearing loss (SNHL) is the most common form of ARHL [10]. It results from pathology of the cochlea or the neural pathway from the cochlea to the brainstem, leading not only to reduced sound audibility but also to distortions in sound perception. These deficits impair speech recognition (particularly in noisy environments) and substantially affect communication, social engagement, and overall well-being [11]. Although HAs effectively improve audibility, they generally have limited effects on maximal speech intelligibility, as measured by the maximum word recognition score (WRSmax), because WRSmax primarily reflects the integrity of the cochlea’s information-carrying capacity rather than audibility per se [12,13,14]. Nevertheless, clinical experience indicates that some patients exhibit measurable improvements in WRSmax following HA fitting, whereas others derive little or no benefit [15,16]. The factors underlying this interindividual variability remain poorly understood, limiting clinicians’ ability to predict rehabilitation outcomes and counsel patients regarding the expected benefits of HA use.

One potential determinant of this variability is audiometric configuration, which reflects the distribution of hearing loss across frequencies and may represent distinct patterns of cochlear pathology. Two common audiometric configurations are the flat type, characterized by relatively uniform hearing loss across frequencies, and the down-sloping (ski-sloping) type, characterized by disproportionately greater high-frequency hearing loss. Because high-frequency hearing contributes importantly to consonant perception and speech discrimination, patients with down-sloping hearing loss may derive greater speech intelligibility benefit from frequency-specific amplification than patients with flat-type hearing loss [17,18]. Targeted amplification of these frequencies by a well-fitted HA may therefore confer a more dramatic perceptual benefit in down-sloping than in flat-type hearing loss [19,20]. However, whether audiometric configuration predicts measurable speech intelligibility benefit after HA fitting has not been systematically investigated.

The present study aimed to determine whether audiometric configuration predicts speech intelligibility outcomes following HA fitting in adults with moderate SNHL. We hypothesized that patients with a down-sloping audiometric configuration would demonstrate greater immediate and cumulative improvements in WRSmax than those with a flat audiometric configuration, supporting the use of audiometric configuration as a practical prognostic marker to guide pre-fitting counseling and patient selection.

## 2. Materials and Methods

### 2.1. Study Design

This retrospective cohort study was conducted at the Department of Otorhinolaryngology, Severance Christian Hospital (Yonsei University College of Medicine, Seoul, Republic of Korea). The study was approved by the Institutional Review Board of Yonsei University (4-2025-0892; approved on 26 August 2025). The requirement for written informed consent was waived because of the retrospective nature of the study. All procedures were conducted in accordance with the ethical principles of the Declaration of Helsinki.

Eligible participants were adults (≥19 years) with newly diagnosed moderate sensorineural hearing loss (SNHL; pure-tone average at 0.5, 1, 2, and 4 kHz [PTA_4_], 40–70 dB HL) who were newly prescribed HAs during outpatient visits between January 2017 and December 2021. Participants were included if they completed at least one audiological follow-up assessment ≥1 month after the initial evaluation. Inclusion criteria were: (1) a new HA recommendation at our institution during the study period; (2) completion of at least one follow-up assessment ≥1 month after the initial evaluation; (3) no previous HA use; and (4) no history of surgical hearing rehabilitation (e.g., cochlear or middle ear implantation). Exclusion criteria were: (1) failure to complete the minimum follow-up assessment; (2) a baseline PTA_4_ threshold >100 dB HL at 2 kHz; (3) an initial difference between aided and unaided speech audiometry scores of <−15 percentage points, suggesting non-organic hearing loss or unreliable test performance; and (4) inability to complete the study assessments. Additionally, for patients using bilateral hearing aids, data from the right and left ears were collected and analyzed separately.

### 2.2. Audiologic Assessment

All participants underwent comprehensive audiological evaluation before HA fitting and at 1-month and 1-year follow-up, including pure-tone audiometry (PTA) and speech audiometry as previously reported [21,22]. PTA thresholds were measured at 250, 500, 1000, 2000, 4000, and 8000 Hz (unaided) and at 250, 500, 1000, 2000, 4000, and 6000 Hz (aided). PTA_4_ was calculated as the mean threshold at 500, 1000, 2000, and 4000 Hz. The word recognition score (WRS) test was conducted for speech audiometry in a quiet environment using a phonetically balanced word list presented at the participants’ most comfortable listening level (MCL). The WRS was expressed as the percentage of correctly identified words, providing an objective measure of speech discrimination ability. The MCL was determined by gradually increasing the intensity of speech stimuli until the participant indicated the most comfortable loudness level for listening, as previously reported [23]. Baseline unaided WRSmax was assessed monaurally using calibrated headphones at each participant’s MCL, with masking noise presented to the contralateral ear at an appropriate level. HA-aided WRSmax at the 1-month and 1-year follow-ups was assessed in a sound-field setting with the loudspeaker positioned at 0° azimuth, at each participant’s aided MCL, while speech masking noise was delivered to the contralateral ear via headphones at an appropriate level.

### 2.3. Audiologic Classification

Participants were classified into two audiometric configuration groups based on their baseline unaided pure-tone audiogram. Audiometric configuration was determined by the difference between the mean higher-frequency hearing threshold (2, 4, and 8 kHz) and the mean lower-frequency hearing threshold (0.25, 0.5, and 1 kHz). Although 1 and 2 kHz are generally considered mid-frequencies, we operationally divided the tested frequencies into these two groups to facilitate a simple classification of audiometric configurations. This approach represents a modification of previously described audiogram classification methods, which categorize hearing loss according to the difference between average low- and high-frequency hearing thresholds [24,25]. Participants with a mean threshold difference of ≥25 dB were classified as having a down-sloping (high-frequency) audiometric configuration, whereas those with a difference of <25 dB were classified as having a flat audiometric configuration. A 25-dB difference corresponds to an average slope of approximately 8.3 dB/octave across the 3-octave span. Although no universally accepted cutoff exists for distinguishing flat from down-sloping audiograms, this operational criterion is consistent with previous approaches that characterize audiometric configuration based on threshold differences across frequencies or the rate of threshold change per octave [26,27]. In the present cohort, which was restricted to participants with moderate sensorineural hearing loss (PTA_4_, 40–70 dB HL), this cutoff was used as a pragmatic operational definition to distinguish a clinically meaningful high-frequency slope from relatively flat audiometric configurations.

### 2.4. Statistical Analysis

Statistical analyses were performed using IBM SPSS Statistics version 27.0 (IBM Corp., Armonk, NY, USA). Graphics were generated using GraphPad Prism 8.0 (Dotmatics, Boston, MA, USA). Baseline characteristics between the down-sloping and flat-type groups were compared using the independent-samples *t* test for continuous variables and the χ^2^ test for categorical variables. A multiple linear regression analysis was conducted to assess the association between hearing loss configuration and WRS improvement, adjusting for age and sex. The five WRSmax-derived indices were compared between groups using the independent-samples *t* test. A two-sided *p* value of <0.05 was considered statistically significant. Variables included in the analysis were audiometric configuration type (flat vs. down-sloping), sex, age, ear side, initial unaided PTA_4_, initial aided PTA_4_, and initial unaided and aided WRSmax. 

## 3. Results

### 3.1. Participant Characteristics

A total of 351 participants met the eligibility criteria and were included in the analysis (Table 1). The mean (SD) age was 70.1 (13.2) years, and 169 participants (48.1%) were male. Based on baseline audiometric configuration, 211 participants (60.1%) were classified as having a flat audiometric configuration and 140 (39.9%) as having a down-sloping audiometric configuration. The mean (SD) baseline unaided PTA_4_ was 55.3 (11.1) dB HL. Baseline demographic and audiologic characteristics according to audiometric configuration are summarized in Figure 1 and Table 2. Patients with down-sloping hearing loss were younger (67.8 vs. 71.6 years, *p* = 0.009) and more likely to be male (62.9% vs. 38.4%, *p* < 0.001), whereas baseline PTA_4_ was comparable between groups. Baseline unaided WRSmax was slightly lower in the down-sloping group than in the flat group, although the difference did not reach statistical significance (53.3% vs. 58.0%; *p* = 0.050).

### 3.2. Short-Term Improvement in WRSmax at 1 Month After HA Fitting

Patients with a down-sloping audiometric configuration experienced greater early improvement in speech intelligibility after HA fitting than those with a flat configuration (Figure 2). The increase in WRSmax from baseline to the 1-month follow-up (1-month aided WRSmax − baseline unaided WRSmax) was significantly greater in the down-sloping group than in the flat group (mean [SD], 10.43 [10.32] vs. 7.18 [9.44] percentage points; *p* = 0.004). This association remained significant after adjustment for age and sex in multiple regression analysis (B = 2.506; *p* = 0.024). However, no significant between-group differences were observed in the absolute aided WRSmax, aided PTA_4_, or MCL at the 1-month follow-up (Table 2).

### 3.3. Long-Term Improvement in WRSmax at 1 Year After HA Fitting

This advantage persisted at the 1-year follow-up (Figure 3). The cumulative improvement in WRSmax from baseline (1-year aided WRSmax − baseline unaided WRSmax) remained significantly greater in patients with a down-sloping configuration than in those with a flat configuration (mean [SD], 11.00 [10.55] vs. 6.79 [11.79] percentage points; *p* = 0.020). After adjustment for age and sex, audiometric configuration remained an independent predictor of greater WRSmax improvement (B = 3.792; *p* = 0.044). Nevertheless, the two groups did not differ significantly in absolute aided WRSmax or MCL at the 1-year follow-up (Table 2).

## 4. Discussion

In this retrospective cohort study of 351 adults with moderate SNHL, patients with a down-sloping audiometric configuration derived greater speech intelligibility benefit from HA rehabilitation than those with a flat audiometric configuration. This advantage was evident as early as the 1-month follow-up and remained significant when cumulative improvement from baseline to the 1-year follow-up was evaluated. Together, these findings support audiometric configuration as a simple and clinically useful predictor of speech intelligibility benefit following HA fitting.

The greater early benefit observed in patients with a down-sloping audiometric configuration is biologically plausible. High-frequency hearing, particularly above 2 kHz, provides critical acoustic cues for consonant perception and speech discrimination [19,20]. In down-sloping SNHL, hearing loss is predominantly concentrated at higher frequencies, allowing frequency-specific amplification by HAs to improve access to high-frequency speech cues that were previously inaudible. By contrast, patients with flat audiometric configurations exhibit relatively uniform hearing loss across frequencies, potentially limiting the relative contribution of high-frequency amplification to speech intelligibility. Accordingly, although amplification improves audibility in both groups, patients with down-sloping hearing loss may experience greater improvements in speech recognition because restoration of high-frequency speech information may have a proportionally greater perceptual impact. This interpretation is further supported by our analysis showing a significant positive correlation between ΔWRSmax and the difference in pure-tone thresholds between higher and lower frequencies, with a stronger correlation observed at the 1-year follow-up than at the 1-month follow-up. These findings suggest that a more steeply down-sloping audiometric configuration may be associated with greater speech-recognition benefit following HA fitting.

Importantly, achieving prescribed target gain may be more difficult at higher frequencies, and real-ear gain can deviate substantially from target gain, particularly above approximately 2 kHz [28]. This consideration may appear to conflict with our finding of greater WRSmax improvement in patients with down-sloping hearing loss. However, to the extent that high-frequency amplification was below target in our cohort, such underamplification would be expected to attenuate, rather than enhance, the speech-recognition benefit in patients with down-sloping hearing loss. The observation of greater WRSmax improvement in this group despite this potential constraint therefore supports the possibility that improved access to high-frequency speech information provides a meaningful perceptual benefit. If prescribed high-frequency gain had been more completely achieved, the difference in speech-recognition benefit between down-sloping and flat audiometric configurations might have been even greater. However, because real-ear measurement (REM) data were not systematically available in this retrospective cohort, this possibility could not be directly evaluated and should be investigated in future prospective studies incorporating standardized fitting protocols and REM.

The greater cumulative improvement observed in the down-sloping group at the 1-year follow-up suggests that the early advantage associated with this audiometric configuration is sustained over time rather than being limited to the initial period after HA fitting. One possible explanation is auditory acclimatization, whereby continued exposure to previously inaudible high-frequency speech cues facilitates more effective processing of speech over time [29,30]. Although the underlying mechanisms were not investigated in this study, enhanced access to consonant-rich high-frequency information may allow patients with down-sloping hearing loss to derive greater cumulative benefit from prolonged HA use.

These findings have important clinical implications. Audiometric configuration is routinely available from standard pure-tone audiometry and therefore represents a simple, inexpensive, and readily accessible prognostic marker. For patients with a down-sloping audiogram, clinicians may reasonably anticipate greater improvements in speech intelligibility following HA fitting and incorporate this information into pre-fitting counseling. Conversely, for patients with a flat audiometric configuration, counseling should emphasize that measurable improvements in speech intelligibility may be more limited despite appropriate amplification. Individualized counseling based on audiometric configuration may help establish realistic expectations, improve adherence to HA use, and potentially reduce premature device abandonment, which remains common in hearing rehabilitation [31,32].

This study has several limitations. First, its retrospective, single-center design may limit the generalizability of the findings. Second, although cumulative outcomes were evaluated over 1 year, longer follow-up is needed to determine whether the observed differences persist over time. Third, HA models and fitting parameters were not standardized across participants, potentially introducing variability in rehabilitation outcomes. Fourth, acoustic coupling parameters—such as dome type (open vs. closed), vent diameter, and custom earmold acoustics—were not controlled. In flat hearing loss, low-frequency acoustic leakage through open venting may have led to low-frequency underamplification, potentially contributing to smaller observed WRS improvements. Finally, several factors known to influence HA benefit, including cognitive function, listening demands, device use, and patient motivation, were not systematically assessed. Prospective multicenter studies with standardized fitting protocols and comprehensive behavioral and cognitive assessments are warranted to validate these findings and further clarify the mechanisms underlying the observed differences.

## 5. Conclusions

In this retrospective cohort study of adults with moderate sensorineural hearing loss, a down-sloping audiometric configuration was associated with greater improvement in speech intelligibility following HA fitting than a flat audiometric configuration. Therefore, audiometric configuration may serve as a pragmatic, preliminary reference during pre-fitting counseling to help establish realistic patient expectations regarding potential speech-recognition benefits.

## Figures and Tables

**Figure 1:**
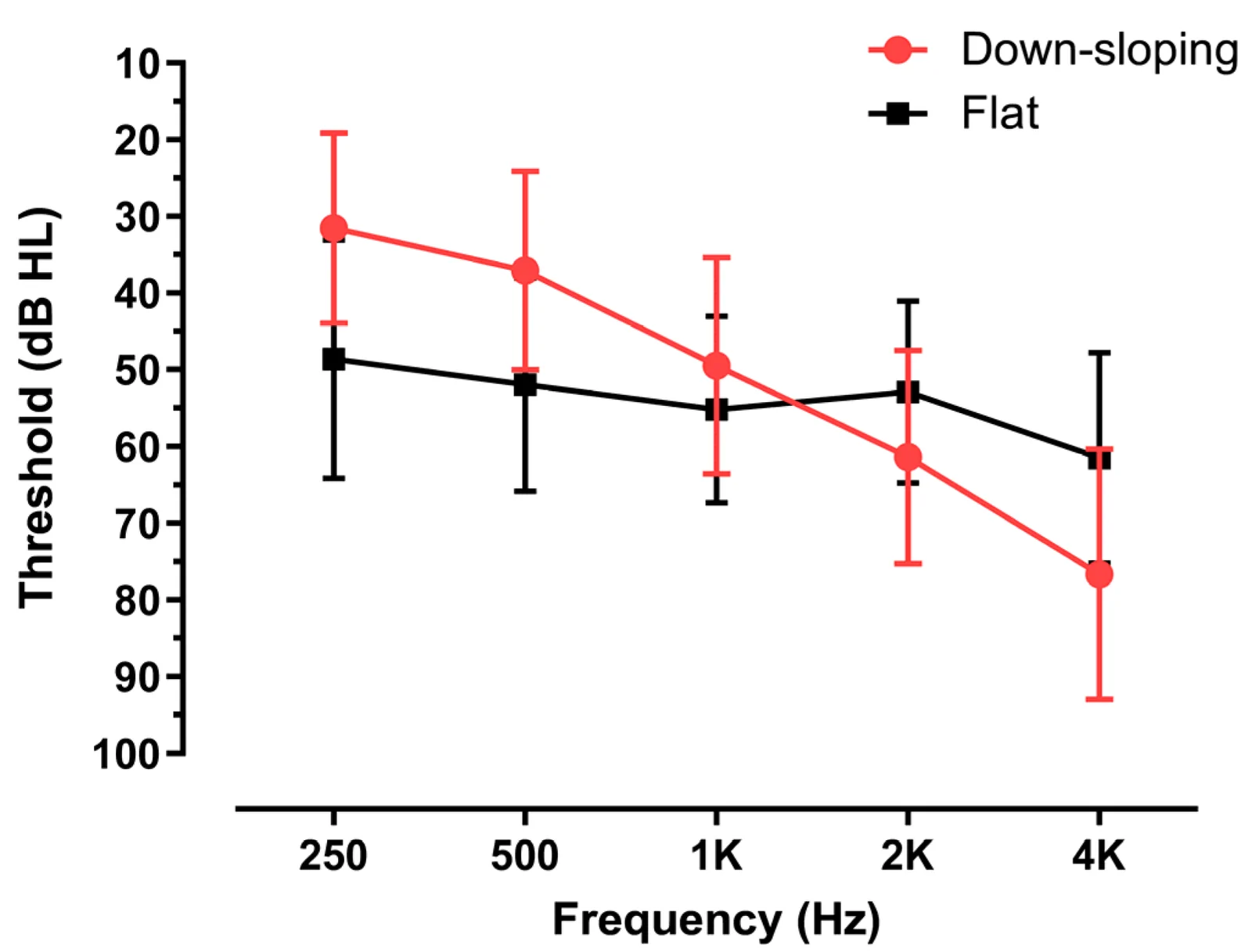
Baseline pure-tone audiograms according to audiometric configuration. Mean pure-tone air-conduction thresholds at each test frequency for participants with flat and down-sloping audiometric configurations. Error bars represent standard deviation (SD). Participants were classified according to the difference between the mean lower-frequency (0.25, 0.5, and 1 kHz) and higher-frequency (2, 4, and 8 kHz) hearing thresholds as described in the methodology section.

**Figure 2:**
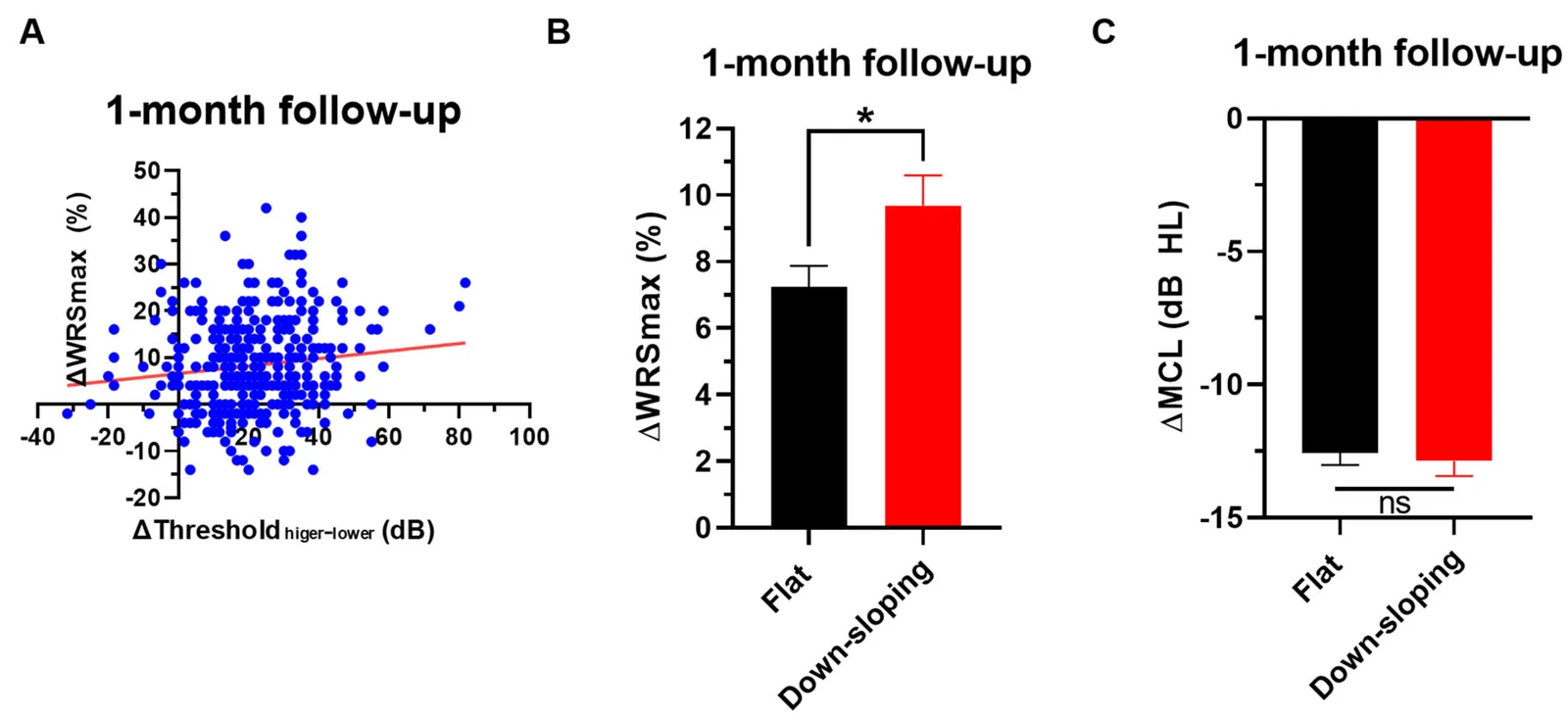
Immediate hearing aid benefit according to audiometric configuration. (**A**) Improvement in maximum word recognition score (ΔWRSmax), calculated as the 1-month aided WRSmax minus the baseline unaided WRSmax, plotted as a function of the difference in pure-tone thresholds between higher frequencies (2, 4, and 8 kHz) and lower frequencies (0.25, 0.5, and 1 kHz). The red line represents the linear regression fit (y = 0.08 × x + 6.567, *p* = 0.0166). (**B**) Comparison of ΔWRSmax between participants with flat and down-sloping audiometric configurations. Participants with a mean threshold difference of ≥25 dB were classified as having a down-sloping (high-frequency) audiometric configuration, whereas those with a difference of <25 dB were classified as having a flat audiometric configuration. (**C**) Change in most comfortable listening level (ΔMCL), calculated as the 1-month aided MCL minus the baseline unaided MCL. Data are presented as mean ± SEM. *, *p* < 0.05; ns, not significant.

**Figure 3:**
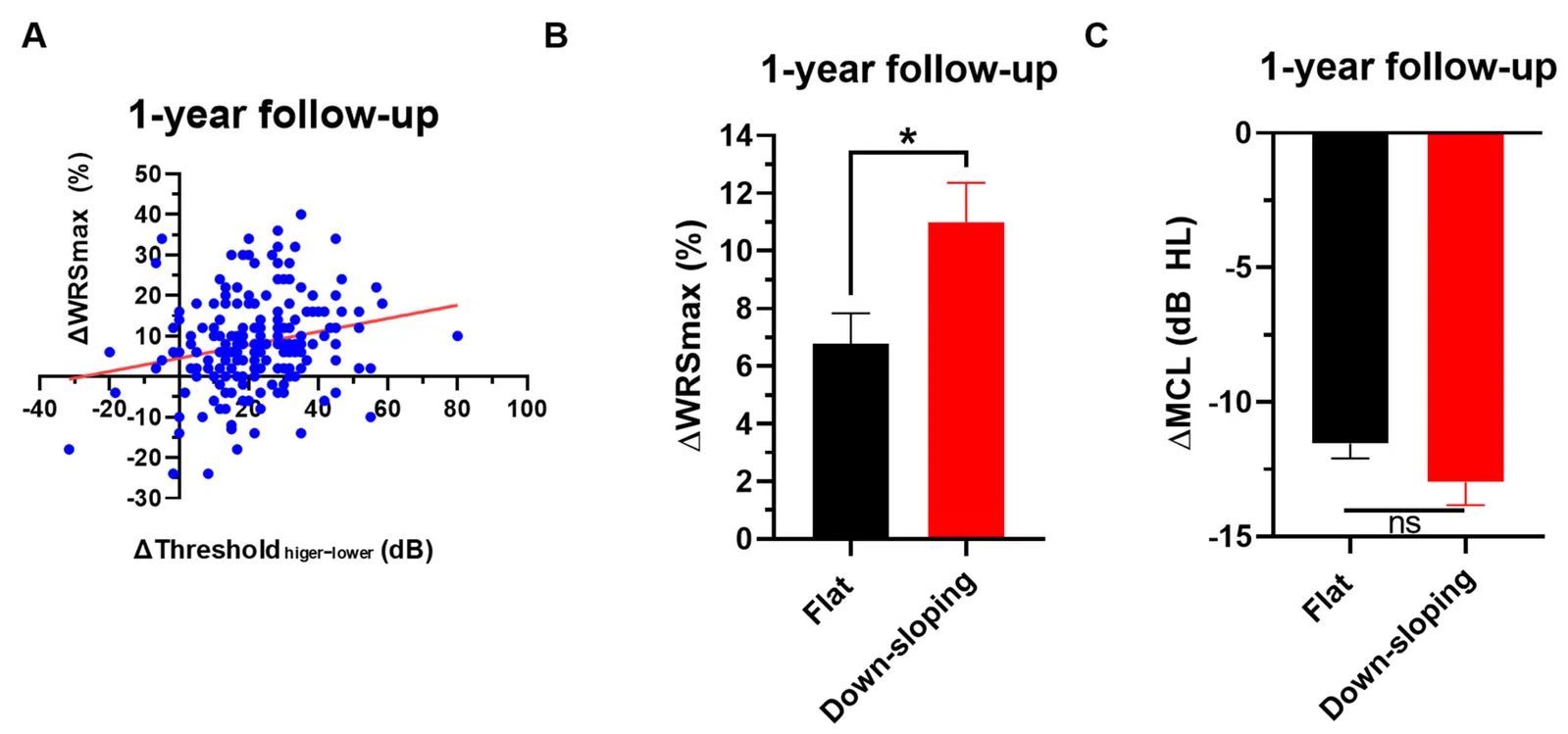
Cumulative hearing aid benefit according to audiometric configuration. (**A**) Improvement in maximum word recognition score (ΔWRSmax), calculated as the 1-year aided WRSmax minus the baseline unaided WRSmax, plotted as a function of the difference in pure-tone thresholds between higher frequencies (2, 4, and 8 kHz) and lower frequencies (0.25, 0.5, and 1 kHz). The red line represents the linear regression fit (y = 0.164 × x + 4.541, *p* = 0.0029). (**B**) Cumulative improvement in maximum word recognition score (ΔWRSmax), calculated as the 1-year aided WRSmax minus the baseline unaided WRSmax, in participants with flat and down-sloping audiometric configurations. Participants with a mean threshold difference of ≥25 dB were classified as having a down-sloping (high-frequency) audiometric configuration, whereas those with a difference of <25 dB were classified as having a flat audiometric configuration. (**C**) Change in most comfortable listening level (ΔMCL), calculated as the 1-year aided MCL minus the baseline unaided MCL. Data are presented as mean ± SEM. *, *p* < 0.05; ns, not significant.

**Table 1:** General characteristics of participants.

Factor	
Age (y)	70.1 ± 13.2
Sex, No. (%)	
Male	169 (48.1)
Female	182 (51.9)
Ear included, No. (Right:Left)	181:170
Audiometric configuration, No. (%)	
Flat type	211 (60.1)
Down-sloping type	140 (39.9)
PTA_4_, dB HL	55.3 ± 11.1
WRSmax, %	56.2 ± 22.2
MCL, dB HL	78.1 ± 8.5
HA-aided PTA_4_ (1 month), dB HL	46.1 ± 9.4
HA-aided WRSmax (1 month), %	64.4 ± 19.9
HA-aided MCL (1 month), dB HL	65.4 ± 6.0
HA-aided PTA_4_ (1 year), dB HL *	44.4 ± 10.8
HA-aided WRSmax (1 year), % *	63.6 ± 21.5
HA-aided MCL (1 year), dB HL *	65.2 ± 5.3

Values are presented as mean ± SD; PTA_4_, four-frequency pure-tone average (0.5, 1, 2, and 4 kHz); HA, hearing aid; WRSmax, maximum word recognition score at the most comfortable listening level; MCL, most comfortable listening level; *, *n* = 186 for patients with 1-year follow-up.

**Table 2:** Comparison of groups with flat versus down-sloping hearing loss (*n* = 351).

Factor	Flat (*n* = 211)	Down-Sloping (*n* = 140)	*p*-Value
Age (years)	71.6 ± 11.9	67.8 ± 14.7	0.009
Sex, No. (%)			<0.001
Male	81 (38.4)	88 (62.9)	
Female	130 (61.6)	52 (37.1)	
Ear included, No. (Right:Left)	113:98	68:72	0.362
PTA_4_, dB HL	54.8 ± 10.8	56.1 ± 11.6	0.278
WRSmax, %	58.0 ± 22.0	53.3 ± 22.2	0.050
MCL, dB HL	78.4 ± 8.2	77.7 ± 8.9	0.436
HA-aided PTA_4_ (1 month), dB HL	45.8 ± 9.0	46.6 ± 10.0	0.426
HA-aided WRSmax (1 month), %	65.3 ± 20.3	63.0 ± 19.4	0.290
HA-aided MCL (1 month), dB HL	65.8 ± 6.1	64.8 ± 5.8	0.114
HA-aided PTA_4_ (1 year), dB HL *	45.1 ± 7.3	43.3 ± 14.5	0.126
HA-aided WRSmax (1 year), % *	64.0 ± 21.9	63.0 ± 21.1	0.228
HA-aided MCL (1 year), dB HL *	65.8 ± 5.0	64.3 ± 5.5	0.413

Values are presented as mean ± SD; PTA_4_, four-frequency pure-tone average (0.5, 1, 2, and 4 kHz); HA, hearing aid; WRSmax, maximum word recognition score at the most comfortable listening level; MCL, most comfortable listening level; *, *n* = 126 and 60 for flat and down-sloping hearing loss, respectively.

## Data Availability

The original contributions presented in this study are included in the article. Further inquiries can be directed to the corresponding authors.
